# Illinois State Dental Society

**Published:** 1886-06

**Authors:** 


### ILLINOIS STATE DENTAL SOCIETY.

---

REPORTED EXPRESSLY FOR THE INDEPENDENT PRACTITIONER.

---

The Illinois State Dental Society convened for its twenty-second annual meeting at Rock Island, Tuesday, May 4, 1886. The sessions were largely attended, and the meeting was one of unusual interest. After the usual preliminary business, the President, Dr. T. L. Gilmer, of Quincy, gave the annual address.

### DISCUSSION.

*Dr. Koch*—There are only two points that I noticed in this address that I think I shall take issue with. If I understood the President correctly, he said that the rank and file of the medical profession at large was constituted of better material than the rank and file of the dental profession.

*The President*—I said better educated.

*Dr. Koch*—I was coming to that—that they were better prepared in the matter of primary education. I doubt that position. I do not know that medical colleges are any more stringent about the preliminary requirements of their students than are the dental colleges Heretofore, I think medical students, as a class, have been made up of about as poor material as dental students, and while they may make greater pretension, there is no foundation for it. The other point to which I wish to refer, is that with reference to recognition by the medical profession. I think we ought to be proud enough of the achievements we have attained not to care very much, neither as individuals or collectively, whether they recognize us or not. I do not think any gentleman in the dental profession will ever fail, as an individual, to receive that recognition he deserves from any intelligent man, medical men inclusive. But when our President states that this recognition will come to any man who has medical education, I take issue with him. I do not think it is based upon medical knowledge, unless that medical knowledge is gained at a certain place, at the medical college; in other words, unless the possessor of that medical knowledge has in his possession a medical degree. A man who is purely a dentist, no matter what his knowlege is, will not receive recognition by the medical profession in its associated condition, without having an M. D. to qualify him for that association. In other words, if our present President had gone to attend the American Medical Association simply with his D. D. S., the doors would not have been opened to him.

*Dr. Noyes*—I think the President makes a distinction which will almost completely reconcile the difference which Dr. Koch has made emphatic. What he has said I believe to be true, in respect to public recognition of us in societies. What the President meant to refer to in his paper was the personal intercourse of professional men, dentists and physicians individually. I believe the positions of both the gentlemen are correct.

*The President*—Dr. Noyes has expressed the sentiments of the paper exactly.

*Dr. Sturgiss*—I listened with some interest to the paper. The ambition that has grown old with the Dental Society of Illinios, to do something that would qualify them for professional recognition by the medical fraternity, seems to be a burden. I am willing to meet the medical fraternity on an equal basis. I am willing to be taxed with as much knowledge of medicine as they have of dentistry. More than that should not be exacted of me. I pledge you my experience that the Dental Society of Illinois to-day knows more about medicine than the Medical Society does about dentistry.

*Dr. Newkirk*—There was one point in the address which struck me—the suggestion as to local societies. Having had a little experience in organizing a local society in this State, I am interested in that subject. Instead of there being but one local society out of Chicago, there ought to be three or four in the State. It is a very easy thing to start one going. All you need is a few men who feel the necessity of it, and who will take hold and assist in organizing. That is what we did when we started the society in Central Illinois. We commenced with five or six members, and at our last meeting in Bloomington, last fall, we had thirty-six.

*Dr. Cushing*—The remarks of Dr. Newkirk suggest to me that the proper way to bring about what he desires, and what we all should desire very heartily, is to appoint from this society a committee with express reference to securing the formation of local societies throughout the State. Those of you who are familiar with the action of the American Dental Association for the last fifteen or twenty years, are perhaps aware that such a committee was appointed from that association, and they visited various sections and aided very largely in stimulating the formation of just such societies, and that is what this society should do. I move that a committee be appointed to devise means for organizing local societies, and to report to this meeting early in its proceedings. The motion was carried.

One of the most interesting and instructive features of the meeting was the presence of Dr. G. V. Black, with his incubator and sterilizing apparatus. During the meeting he made over forty cultures of the micro-organisms of the oral cavity, and at each session he exhibited the progress made and explained the changes as illustrated by the cultures which he had made. At this point in the proceedings he made his first report.

MICRO-ORGANISMS.

*Dr. Black*—About all that I will do now will be to explain the apparatus for the culture of micro-organisms, as near as I can, by the apparatus which I have here.

The crust of the earth is composed of about sixty-five simple substances. Each one of these has a special affinity for other substances, and the play of these affinities gives us inorganic chemistry. The chemical forces, as such, tend to stability, and the combinations made will stand through all eternity if not disturbed. These are changed, are disturbed by light, heat, electricity, storm, wind, etc., and we still have inorganic chemistry. The other disturbing force is life, which gives us organic chemistry. In the apparatus, which I will explain, we propose to study some of the influences of life; and let me say that life is all the same, whether it is in the microbe or in the man. The man or the animal by remoleculization of food produces urea, the higher plants alkaloids, and some of those in which we are interested, lactic acid. This apparatus is for the purpose of bringing out what we can of these changes produced by micro-organisms that grow in the human mouth, that grow in the places where we work, and certainly we should familiarize ourselves with them.

This matter of the culture of micro-organisms has been looked upon by most people in the profession, and out of it, as something far away, that could be done only by some men especially trained from their youth up, who have to be wonderfully careful and sagacious (and after all that, he is very uncertain as to what he may or may not have discovered), and that it is of no use for an ordinary man to look into it. I simply want to say that this is not the case, that there is nothing so wonderful about the matter; it is really very simple, and there are plenty of men in this society who can undertake this work and do it well, and I bring this apparatus here at this time with the hope of inducing some young men, who have the time and ability, to take hold of this subject. The work does consume an immense amount of time, and whoever undertakes it should have the ability to control his leisure.

He then exhibited to the society his apparatus for the culture of micro-organisms, and explained its construction, and also the method of sterilization and planting.

AFTERNOON SESSION.

The report on Dental Science and Literature was made by Dr. C. R. E. Koch, of Chicago.

*Dr. Black*—One point upon which I wish to speak is that with regard to peridental membrane. I shall speak only from my own knowledge. It seemes to me there can be no question as to the character of this membrane. It is not a periosteum at all. It has not the characteristics of periosteum, except that it has a covering on one side. It is not composed as is the periosteum; does not look like it. I certainly have been able to trace fibers from the tooth to the alveolus directly, the one from the other, reaching the whole distance. It does not behave like a periosteum in inflammations, either. It is very remarkable to me that it should still be considered a periosteum by so many. The fibers are placed in a direct position in relation to the tooth and its alveolus, always in the same position, running obliquely from the one to the other, always inward from the alveolus towards the root and towards its apex, throughout the whole body of the root of the tooth, while the fibrous tissues of the periosteum is a net work. We have none of that net work in the peridental membrane. Certainly, it is faulty examination that brings about such an idea as this.

I have been struck with the general want of information in our profession as to what has been done in years gone by. We speak of ourselves as though we had just got out of the shell—a very young profession. That is not true. We are almost as old as the medical profession. The fact is, there was not a dental profession separate and apart from the medical profession until the time of Harris. It was the action of Harris and two or three others, who were influential in his time, that brought about this separation. They lectured in medical schools, were frowned upon, and went out and made a school of their own, and we have been a distinct profession ever since.

*Dr. Talbot*—Do you mean to say that the peridental membrane is not connected with the periosteal membrane?

*Dr. Black*—I say there is no periosteal membrane there.

*Dr. Talbot*—How is it that when we extract a tooth we bring away the periosteal membrane ?

*Dr. Black*—We do not. We tear the peridental membrane at about its middle. It is richly filled with blood vessels traversing it from the apex to the root, and we tear that midway. When we have an abscess the peridental membrane is not parted from the root of the tooth. The fiber is lengthened and the pus nestles among the fibers, and then when we pull out that root we pull out a great sac. In extracting the tooth we tear those fibers in two.

The doctor here illustrated upon the blackboard the position of the various parts referred to.

*Dr. Cushing*—Do I understand you to say that the membrane has not the function of the periosteum?

*Dr. Black*—I did not say that. Those fibers pass directly into the cementum.

*Dr. Cushing*—Does it have the function of the periosteum?

*Dr. Black*—It is in the osteoblasts that the function of the periosteum exists, so far as the bone-forming is concerned.

*Dr. Cushing*—Are not the osteoblasts a part of this membrane, as much as they are a part of the periosteum?

*Dr. Black*—Just as much as they are a part of the periosteum, but they are not a part of either. The difference is not in the supply of blood, which is one of the functions of the periosteum, and it is not in the fact that there are osteoblasts in both cases, but it is in the character of the tissue of which these two members are made up. As far as function is concerned, it is the same, but we do not want to confound two distinct things in this way in histology. They are entirely distinct in character. While one simply covers the bone, the other holds the tooth in its position.

*Dr. Koch*—The line which the doctor draws through the center, as shown upon the board, he says is a blood-vessel. I have no doubt he knows, but I have seen that same picture drawn a little differently. The fibrous condition is represented, and then there is a line that is supposed to be a line of demarcation. On the other side the fibers are articulated, supposed to pertain to the structure of the pericementum, running entirely different, and Dr. Ingersoll stated in his paper that he called on Dr. Black, and he cites Dr. Black's slides to prove his own theory. He said he saw it in one of Dr. Black's preparations—saw the line of demarcation—and I presume that is the picture which I saw. I do not think we ought to take Dr. Black's word for it when there are other persons who believe differently, and I hope some one will examine further and either confirm or disprove it.

*Dr. Talbot*—I agree with Dr. Ingersoll in regard to that matter, although I have not had very much experience in making experiments. When we extract a tooth we can split that membrane in the middle, and that fibers and blood-vessels run through the center seems to me impossible. Having one style of structure on one side, the alveola process and the root of the tooth on the other, to a certain extent it is essential that we should have a varied membrane between the two structures.

*Dr. Noyes*—This seems to be a theoretical question entirely. It seems to me the question of tearing the membrane would depend entirely upon whether the fastenings at the ends of the ropes were stronger than the ropes themselves.

*Dr. Wassall*—I should like a little light from Dr. Black as to the functions of this membrane in question. The membrane has on one side cementum, and we are taught that the cementum is augmented, divided from this membrane; and on the other side of the membrane we have the alveolar wall. We are also taught that the alveolar wall is augmented and increased or absorbed by this membrane. I believe it is practically one membrane, but I should like to know how we can reconcile two functions with one membrane.

*Dr. Black*—I would say that this matter has been under discussion from my earliest acquaintance with histologic subjects. I

remember that Dr. Atkinson made a speech in 1867, in which he maintained stoutly that a membrane could not "functionate" two ways at one time; therefore it was a dual membrane. That is proof positive. There is misconception as to the office or function of the periosteum. Periosteum does not build bone.

*Dr. Wassall*—Sometimes it does.

*Dr. Black*—Not at all. Osteoblasts do. But osteoblasts are not periosteum.

*Dr. Taylor*—Are they separate and distinct?

*Dr. Black*—They are as separate and distinct as the muscle that is attached to the periosteum is from the periosteum.

*Dr. Taylor*—The osteoblasts are not, then, a part of the periosteum?

*Dr. Black*—They are not a part of the periosteum.

*Dr. Cushing*—Do you have osteoblasts without the periosteum?

*Dr. Black*—Certainly; we have osteoblasts all the time without periosteum. We have osteoblasts everywhere through the bone. Periosteum does not go all through.

*Dr. Cushing*—It does on the periphery.

*Dr. Black*—We have osteoblasts under the attachment of ligament where there is no periosteum, and it is these cells that have the function of bone-forming, not the periosteum stripped of these cells. It has been a great misconception. It is altogether wrong. The periosteum is not a productive membrane. It is a fascia, and has about the same office in relation to the bone that the fascia has to the muscle; not a bone-builder, if you separate it from the osteoblasts that lie on its inner surface. That is the fact about the matter.

*Dr. Davis*—Are not the osteoblasts nourished from the periosteum?

*Dr. Black*—They are nourished from the blood supply, and that blood supply does not depend particularly upon the periosteum. It is true that the periosteum does carry a large amount of blood supply in the inner half, the half next the bone, and in this way becomes a depot of supply to the bone, and you may destroy the bone by cutting off its blood supply, not because the periosteum is gone. If you could get the blood supply from some other direction it would not die.

*Dr. Brophy*—I believe that the pericementum is the medium through which nourishment is transmitted from the bone, or from the nutrient vessels, for the building up of cementum, but it does not have anything to do with the nourishment of the bone which forms the socket around the tooth. I believe the bone receives its nourishment from without, and that this membrane is simply devised by nature for the purpose of supplying the teeth with the nourishment necessary for it maintenance. This view is not based upon research, but it is derived in a general way by a course of reasoning, without having studied it critically, as it has been studied by Dr. Black. I think the osteoblasts which lie beneath it are intended for the purpose of supplying the bone with nourishment, and, as he said, it can be demonstrated in various parts of the bony structure where periosteum does not exist.

*Dr. Black*—The tissue of the peridental membrane forms the junction between the tooth and its socket. That is its function. Within that tissue we have nerves of touch, and there we find the sense of touch. They also support blood-vessels which carry blood for the maintenance of surrounding structures.

*Dr. Taylor*—I would like to know whether that has any function so far as the alveolar process is concerned, or is it solely for the support and sustenance of the teeth?

*Dr. Black*—Simply to hold the tooth in its place.

*Dr. Harlan*—I want to say a word or two about dental science, which includes a consideration of the nature of the alveolar dental membrane. Dental science, as I understand it, is not limited to a consideration of works devoted strictly to dental surgery, or practice, nor even physiology. We have had during the past year a great many contributions in different countries to the practical study of bacteriology, and while we are having demonstrations here I wish to call the attention of members of the society to Kline's work on micro-organisms, and to Hueppe's Bacteriological Investigation, and to recall the attention of the society to the work of Sternberg, as I think it will be of benefit to the majority of those members who have not already read those works to get them and learn the A B C of bacteriology. I would not omit, in this connection, to refer to Cruikshank, although his is not the very latest work in that list. Those of us who may purchase or have access to nearly all the new works sometimes think, in looking over them, that there is no real advance, and so we are looking for something else.

But the body of practitioners do not, perhaps, even get the primers, and it is their duty, on account of the magnitude and importance of the germ theory of disease, that they should study works of this character, and those also of a more practical nature.

WEDNESDAY MORNING'S SESSION.

Dr. Black showed some cultures of bacteria made from infections of the day before. The first was a gelatine producing fungus very frequently found in the mouth. Others were found under a plate worn in the mouth. Dr. Black stated that some form of fungus was almost invariably found under artificial plates, and under rubber and celluloid bases the fungi were usually found in great numbers. Of these he exhibited two—*Streptococcus magnus* and *S. continuosus*. Some of them showed a very powerful acid reaction. Dr. Black stated that unless great care was taken to keep a plate clean, these fungi would be found in masses, and much of the sore mouth that was common in the wearing of artificial teeth was doubtless due to these fungi. He stated that his observations completely confirmed those of Dr. Miller, but he has ventured on giving to the different varieties names which he thought appropriate, while Dr. Miller was content to designate the varieties discovered by him through numbers. Dr. Black showed a fungus which he believed identical with the "Delta" variety of Miller.

The *Streptococci* have many species of different sizes. *S. magnus* grows under artificial plates, but was not a fungus of dental caries, as it is too large to enter the dental tubuli. It produces a very powerful acid, and is undoubtedly active in the dissolving of the teeth at the edges of partial plates, by which they are sometimes almost cut through.

A gelatine forming fungus exhibited, grows only in an alkaline medium. The solution does not, however, remain alkaline, although the gelatine formation may continue. He subsequently exhibited a culture tube nearly filled with gelatine, which was produced by the fungus living in a beef-extract-sugar solution, and which was neutralized whenever it became acid.

Dr. A. W. Harlan, of Chicago, read a paper entitled

ANTISEPTICS AND DISINFECTANTS.

Antiseptics, he said, have been used by dentists without a desire to know why they are employed, or the manner in which they act.

They have simply looked at results, and not at methods. We should comprehend the causes which render antiseptices, germicides, disinfectants and deodorizers necessary. The germ theory of disease is now accepted among pathologists, and it is responsible for the introduction of a new class of drugs.

Antiseptics and disinfectants are indicated when we wish to restrain putrefaction, or remove its results. Germicides are also needed to destroy the agents which are essential to decomposition. Antiseptic dressings are indicated in all surgical operations; as much in dental as in general surgery. The hands, instruments, syringes and every appliance, should be cleansed with an antiseptic dressing, sufficiently potent to make inert any septic matter adhering to them.

It requires judgment and knowledge to select the proper drug. When drilling for the first time into a dead tooth, one might not think it essential to use great precaution, yet if antiseptic measures are adopted there will be a saving of time which alone will repay all the care exercised. If judiciously employed, they may prevent a long treatment for periostitis. In such cases it will no longer answer to say that the patient "has caught cold." That excuse may answer for ignorant persons, but it will not do for those who are educated. It is dangerous to use a probe in the canal of a dead tooth, without first disinfecting the debris. To push a broach through the end of a root at the first sitting is almost sure to result in trouble. To cleanse a root and fill it at one sitting is reckless folly, if it be a blind abscess without a fistulous opening.

The indications for the use of antiseptics lie in engorged antrums, in septic roots of teeth, in carious and necrotic tissues, in diseased mucus membrane or gums. Wherever there is a foul smell or decomposing matter, we should at once remove and purify. Disinfectants and antiseptics are employed for quite different purposes. As it is impossible to use great heat in the mouth to destroy the infective spores, we must use something else, and hence germicides are necessary. Germs and spores are found in every part of the mouth, and throughout the whole alimentary tract. They are in the air, and ready to find a lodgment in any weakened tissue. These must be destroyed. Some antiseptics will not destroy germs without causing inflammation of living tissues; others will destroy germs, but not spores. Some antiseptics are not disinfectants, and *vice versa.* Deodorizers may be neither of the other two, and germicides may neither disinfect nor deodorize. What we need is knowledge as to just which of these classes is required, and to what class any remedy belongs. It is my aim to use such drugs as will best perform the duty demanded, and with the least tendency to inflammation, and such as are the least obnoxious to taste or smell, or injurious to instruments.

What is an antiseptic? It is an agent which will prevent decomposition; which will arrest it when begun; which is opposed to putrefaction.

What is a disinfectant? An agent which will destroy foul odors by combining with them chemically; which will not coagulate the surface and leave the interior to putresce; which will cleanse and purify and destroy infection.

What is a germicide? An agent that will destroy germs and their spores, either directly or indirectly. Heat and cold are of course excluded, for neither can be used in the mouth.

In choosing an antiseptic the object sought for should be steadily kept in mind. If we wish to seal a cavity in a tooth containing a living pulp, we should not choose alcohol, permanganate of potassium, or a disinfecting fluid, but rather carbolic acid, aseptol, creosote, terebin, resorcin, iodol, iodoform, menthol, eugenol, eucalyptol, sanitas oil, hydronapthol, thymol, napthol, boro-glyceride, or some other strictly antiseptic agent. If we wish to disinfect a root, we should use Labarraque's solution of chloride of sodium, Condry's fluid, an aqueous solution of chloride of zinc, sanitas fluid No. 1, per-oxide of hydrogen, solutions of aluminum, acetate or chloride, carbon disulphide, corrosive sublimate, biniodide of mercury, hypochloride of calcium, or sodium, iodine, resorcin, boracic acid, or benzoic acid as disinfectants, for they would permeate the whole mass of putrid matter, and not coagulate the surface or substitute the odor of the drug for that which it was desired to disinfect. The object of chemical disinfection in dental surgery is to remove foul odors, destroy the agents of infection, and cleanse the parts to which the agent is applied. If a germicide be needed it is then applied. The part should now be dressed antiseptically, and all debris and foreign matter excluded.

We should not use antiseptics that are very volatile, or poisonous, or disagreeable. Some agents are much more powerful than others, and some germs withstand antiseptics much more than others. Some antiseptics act by precipitating organic matter, and thus they starve organisms. In disinfecting it must be remembered that all infectious material is not foul-smelling, and the absence of odor is not always evidence of decomposition. Many commercial disinfectants are of little value, and some of those most loudly advertised are the most worthless.

The essayist presented various tables, showing the expense and comparative value of different preparations for disinfectant purposes, corrosive sublimate being used as the standard of comparison. He concluded that a solution of sublimate is the most powerful and economical of all the disinfecting preparations. It would require, to perfectly disinfect a room having a capacity of twelve cubic feet, no less than seventeen pounds of pure carbolic acid crystals. Hence it should never be used for this purpose, but only as an antiseptic. Nor should we select a drug because its odor overcomes and drowns the smell of putrefaction, which it has not neutralized at all. It requires a knowledge of the agents to properly select that which is best.

The discussion of the paper was by vote postponed until another session, to allow the introduction of some business matters.

Wednesday afternoon was devoted to clinics.

### WEDNESDAY EVENING SESSION.

Prof. W. D. Miller, of Berlin, was elected an honorary member of the Illinois State Society by acclamation.

The committee appointed to devise means for the establishment of local societies throughout the State, recommended the appointment of a committee of seven members as a central or governing committee, whose duty it shall be to secure the formation of six subcommittees from dentists outside the Illinois State Society, to act for six districts, to be subsequently formed.

The Committee on Dental Art and Mechanism reported, through their chairman, Dr. J. Frank Marriner. A number of new and original devices were presented, some of them evidently of permanent value, together with some new methods of practice. The large number of these devices and modifications indicates great inventive genius on the part of members. It was impossible to obtain a complete list and description of them, and hence individual mention is omitted.

Dr. Black was called upon for a report upon his cultures, and said:

Before speaking particularly of the cultures I will demonstrate that the acids of the mouth are due to these fungi. The saliva when secreted is seldom acid, but it soon becomes so. I will take a drop from this culture tube containing a common fungus of the mouth, and will touch with it this piece of litmus. This is not a fungus of caries, but obtained from the dorsum of the tongue. You see that the culture fluid is intensely acid.

There are many ways of obtaining pure cultures. In such a room as this, where so many organisms are floating about, it is difficult to obtain clean cultures. The tubes become infected with strange organisms. If we have, however, two forms, say of *Streptococcus*, which we wish to separate, we may do it by their specific gravity. While one settles rapidly, the other will do so but slowly. Sometimes in this way we may get a pure culture the first day. One form grows rapidly and another slowly. Others will not separate at all, and we must employ the decimal plan. By means of a platinum wire previously heated to redness, I remove to a solution in a previously sterilized tube a single drop of any infection, and stir it up well. We may now take a drop of this fluid and transfer it to another tube containing a hundred drops of distilled water, and after shaking, examine a drop with the microscope. If the examination be not satisfactory, we may continue the dilution in another tube. Dr. Black also described gelatine culture plates, and other forms of cultures. He dilated upon the necessity for careful preliminary sterilization, and care to avoid foreign infection. When it is desired to grow any fungi, the general principles are those of gardening. A suitable soil must be procured and all weeds kept out. Then if the whole be kept at a proper temperature, the fungi will grow very rapidly. The most effective way is by culture plates, which are planted and then kept in the incubator. Dr. Black proceeded at some length to describe other methods of obtaining pure cultures, illustrating them by his apparatus, which was present. He paid a glowing tribute to Dr. W. D. Miller, whose scientific work in this direction stood for a model to the world. Others were but following where Dr. Miller led.

*Dr. H. J. McKellops*—Asked for information concerning different stages of decay.

*Dr. Black*—Said that some time ago we were all making examinations of the saliva with litmus. We found that immediately after a meal the saliva was alkaline or neutral, but in a few hours it became acid. This is because the great flow of saliva during the ingestion of a meal washes out the fungi and their products, but in a little time they again proliferate, and we find the fluids of the mouth acid. Hence the testing of the fluids of the mouth is but child's play, and teaches nothing whatever, of itself. Caries is not necessarily a continuous operation. The fungi die and another kind may succeed them.

*Dr. McKellops*—Often a mouth will go for years without decay, when all at once a kind of white caries will attack it with extraordinary violence.

*Dr. Black*—I recognize all this, and I live in the hope that in time we shall be able to control caries by systemic and topical treatment. During the year spoken of the secretions of the mouth were in a condition unfavorable to the growth of the fungi of decay. All at once, perhaps, these circumstances changed, and the fungi multiplied prodigiously. If we can control these conditions we can control decay. The fungi are very sensitive to unfavorable conditions. At one time I allowed my incubator to get cold, when every fungus died.

*Dr. Templeton* — Have you made cultures of fungi from the mouth before eating, to see if they be of a different character? Also, have you made special examinations of the fungi during gestation and lactation?

*Dr. Black*—I have not got that far yet. The question shows how great is the field yet unexamined. We know that there are different manifestations of decay at different periods, and there must be different forms of organisms, or a difference in their proliferation.

*Dr. Reid*—In neutralizing the fluids, do you do it by exact measure, or by trial?

*Dr. Black*—By trial, always. If the fluid contained always a definite amount of acid, we might use a definite amount of alkali; but this is not the case, and we must continue to carefully add the neutralizer until it is no longer acid.

(TO BE CONTINUED.)

